# The role of α_1_- and α_2_-adrenoceptor subtypes in the vasopressor responses induced by dihydroergotamine in ritanserin-pretreated pithed rats

**DOI:** 10.1186/s10194-017-0812-4

**Published:** 2017-10-11

**Authors:** Eduardo Rivera-Mancilla, Victor H. Avilés-Rosas, Guadalupe Manrique-Maldonado, Alain H. Altamirano-Espinoza, Belinda Villanueva-Castillo, Antoinette MaassenVanDenBrink, Carlos M. Villalón

**Affiliations:** 1 Department of Pharmacobiology, Cinvestav-Coapa, Czda. de los Tenorios 235, Col. Granjas-Coapa, Deleg. Tlalpan, C.P, 14330 Mexico City, Mexico; 2 Division of Vascular Medicine and Pharmacology, Erasmus University Medical Center, P.O. Box 2040, 3000 CA Rotterdam, The Netherlands

**Keywords:** α-Adrenoceptors, Dihydroergotamine, Pithed rat, Ritanserin, Vasopressor responses

## Abstract

**Background:**

Dihydroergotamine (DHE) is an acute antimigraine agent that displays affinity for dopamine D_2_-like receptors, serotonin 5-HT_1/2_ receptors and α_1_/α_2_-adrenoceptors. Since activation of vascular α_1_/α_2_-adrenoceptors results in systemic vasopressor responses, the purpose of this study was to investigate the specific role of α_1_- and α_2_-adrenoceptors mediating DHE-induced vasopressor responses using several antagonists for these receptors.

**Methods:**

For this purpose, 135 male Wistar rats were pithed and divided into 35 control and 100 pretreated i.v. with ritanserin (100 μg/kg; to exclude the 5-HT_2_ receptor-mediated systemic vasoconstriction). Then, the vasopressor responses to i.v. DHE (1–3100 μg/kg, given cumulatively) were determined after i.v. administration of some α_1_/α_2_-adrenoceptor antagonists.

**Results:**

In control animals (without ritanserin pretreatment), the vasopressor responses to DHE were: (i) unaffected after prazosin (α_1_; 30 μg/kg); (ii) slightly, but significantly, blocked after rauwolscine (α_2_; 300 μg/kg); and (iii) markedly blocked after prazosin (30 μg/kg) plus rauwolscine (300 μg/kg). In contrast, after pretreatment with ritanserin, the vasopressor responses to DHE were: (i) attenuated after prazosin (α_1_; 10 and 30 μg/kg) or rauwolscine (α_2_; 100 and 300 μg/kg); (ii) markedly blocked after prazosin (30 μg/kg) plus rauwolscine (300 μg/kg); (iii) attenuated after 5-methylurapidil (α_1A_; 30–100 μg/kg), L-765,314 (α_1B_; 100 μg/kg), BMY 7378 (α_1D_; 30–100 μg/kg), BRL44408 (α_2A_; 100–300 μg/kg), imiloxan (α_2B_; 1000–3000 μg/kg) or JP-1302 (α_2C_; 1000 μg/kg); and (iv) unaffected after the corresponding vehicles (1 ml/kg).

**Conclusion:**

These results suggest that the DHE-induced vasopressor responses in ritanserin-pretreated pithed rats are mediated by α_1_- (probably α_1A_, α_1B_ and α_1D_) and α_2_- (probably α_2A_, α_2B_ and α_2C_) adrenoceptors. These findings could shed light on the pharmacological profile of the vascular side effects (i.e. systemic vasoconstriction) produced by DHE and may lead to the development of more selective antimigraine drugs devoid vascular side effects.

## Background

Both ergotamine and dihydroergotamine (DHE) share structural similarities with serotonin, dopamine and (nor)adrenaline, and have been shown: (i) to display affinity for a wide variety of receptors including serotonin 5-HT_1/2_, dopamine D_2_-like and α_1_/α_2_-adrenoceptors [1]; and (ii) to be effective in the acute treatment of migraine [2]. Nevertheless, unlike ergotamine, DHE (a hydrogenated ergot synthesized by reducing an unsaturated bond in ergotamine) displays a much lower vasoconstrictor and emetic potential [1]. Indeed, recent pharmaceutical developments have introduced the use of inhalable DHE (iDHE) in nasal sprays and oral inhalers; these novel iDHE medications are better tolerated than i.v. DHE and provide an important option for the acute therapy of migraine [3–5].

Regarding the systemic vasoconstrictor potential of DHE, Roquebert and Grenié [6] have reported that DHE produces vasopressor responses in pithed rats by activation of α_2_ (but not α_1_) adrenoceptors, since such responses were: (i) attenuated by 500 and 1000 μg/kg yohimbine (an α_2_-adrenoceptor antagonist); and (ii) apparently resistant to blockade by 500 μg/kg prazosin (an α_1_-adrenoceptor antagonist). At that time, however, it was unknown that: (i) DHE also displays a high affinity for 5-HT_2A_ receptors (pK_i_ = 8.54) [7], whose activation in resistance blood vessels induces vasopressor responses [8] that may have masked the capability of DHE to activate α_1_-adrenoceptors; and (ii) yohimbine displays a moderate affinity for α_1_-adrenoceptors (pK_i_ = 6.6) [9].

Since, in addition, DHE can interact with all α_1_/α_2_-adrenoceptor subtypes (see Table 1), all of the above findings taken together raise the question whether systemic 5-HT_2A_ receptor blockade would unmask the role of α_1_-adrenoceptors and, consequently, the capability of prazosin to block the vasopressor responses to DHE. Interestingly, ergotamine produces vasopressor responses in pithed rats via the activation of α_1A_, α_1B_, α_1D_, α_2A_ and α_2C_ (but not α_2B_)-adrenoceptor subtypes [10], but no study has yet reported the specific role of these subtypes in DHE-induced vasopressor responses in pithed rats. This is an experimental model predictive of systemic (cardio)vascular side effects [11, 12]; since this model is devoid of a functional central nervous system (see General methods below), one can categorically exclude the compensatory baroreflex mechanisms typically observed in intact or anaesthetized animals.

**Table 1:** Binding affinity constants (pK_i_) of the drugs used in this study for α_1_- and α_2_-adrenoceptors

Drug	α_1_			α_2_		
Ritanserin	6.7^1,b^			6.2^1,b^		
Drugs	α_1A_	α_1B_	α_1D_	α_2A_	α_2B_	α_2C_
5-Methylurapidil	9.0^2,b^	7.4^2,b^	7.6^2,b^	6.2^3^	6.4^3^	6.9^3^
L-765,314	6.3^4,b^	8.3^4,b^	7.3^4,b^	N.D.	N.D.	N.D.
BMY 7378	7.1^2,b^	7.5^2,b^	9.0^2,b^	5.1^5,b^	5.1^5,b^	5.1^5,b^
BRL44408	N.D.	N.D.	N.D.	8.7^6,b^	6.9^6,b^	6.8^7^
Imiloxan	< 4^8^	< 4^8^	< 4^8^	5.5^8^	7.3^8,b^	6.0^9^
JP-1302	N.D.	N.D.	N.D.	5.5^10^	5.8^10^	7.6^10^
Prazosin	9.5^11,b^	9.7^11,b^	9.6^11,b^	5.6^12^	6.9^12^	7.2^12^
Rauwolscine	5.3^13^	5.9^13^	6.4^13^	8.4^12^	8.3^12^	9.1^12^
Dihydroergotamine	8.6^14,b,a^	8.0^14,b,a^	7.8^14,b,a^	8.7^15^	8.0^15^	9.0^15^

Data taken from the following references: ^1^ [37]; ^2^ [38]; ^3^ [39]; ^4^ [40]; ^5^ [41]; ^6^ [42]; ^7^ [43]; ^8^ [44]; ^9^ [45]; ^10^ [46]; ^11^ [47]; ^12^ [48]; ^13^ [49]; ^14^ [50]; and ^15^ [7]

All values have been presented as pK_i_, except for: ^a^ pA_2_

^b^ Values for rodent receptors

N.D. stands for “not determined”

Based on the above findings and using antagonists with relative selectivity for α_1_- and α_2_-adrenoceptors (Table 1) at blocking doses (see below) in pithed rats [10, 13], the present study has re-investigated the vasopressor responses to DHE in an attempt to: (i) analyze the specific role of α_1_- and α_2_-adrenoceptors in control animals; and (ii) ascertain the possible involvement of their corresponding subtypes in animals pretreated with ritanserin (100 μg/kg, i.v.). Ritanserin is an antagonist with a very high affinity for 5-HT_2A_ receptors (pK_i_ = 9.5) [14] and very low affinity for α_1_- and α_2_-adrenoceptors (see Table 1) that, in pithed rats: (i) is devoid of α_1_-adrenoceptor blocking properties (up to 3000 μg/kg, i.v.) on the vasopressor responses to phenylephrine [15]; and (ii) practically abolishes (at 30 μg/kg, i.v.) the cardiovascular responses mediated by 5-HT_2A_ receptors [16].

## Methods

### Animals

Experiments were carried out in 135 male normotensive Wistar rats (250–300 g, 8 weeks of age). The animals were housed in a special room at a constant temperature (22 ± 2 °C) and humidity (50%), and maintained at a 12/12-h light/dark cycle (light beginning at 7:00 am), with ad libitum access to food and water. The animal procedures, the experimental protocols and number of animals used in this investigation were reviewed and approved by our Institutional Ethics Committee on the use of animals in scientific experiments (CICUAL Cinvestav; protocol number 507–12) and followed the regulations established by the Mexican Official Norm (NOM-062-ZOO-1999), in accordance with the ARRIVE (Animal Research: Reporting In Vivo Experiments) reporting guidelines for the care and use of laboratory animals.

### General methods

After anaesthesia with sodium pentobarbital (60 mg/kg, i.p.) and cannulation of the trachea, the 135 rats were pithed by inserting a stainless steel rod through the orbit and foramen magnum, and down the vertebral foramen [17]. Then, the animals were artificially ventilated with room air using a model 7025 Ugo Basile pump (56 strokes/min.; stroke volume: 20 ml/kg) as previously established [18]. After cervical bilateral vagotomy, catheters were placed in: (i) the left and right femoral veins for i.v. bolus injections of DHE or antagonists, respectively; and (ii) the left carotid artery, connected to a Grass pressure transducer (P23XL), for recording arterial blood pressure. Heart rate was measured with a tachograph (7P4, Grass Instrument Co., Quincy, MA, USA), triggered from the blood pressure signal. Both parameters were recorded by a model 7 Grass polygraph (Grass Instrument Co., Quincy, MA, USA).

**Fig. 1:**
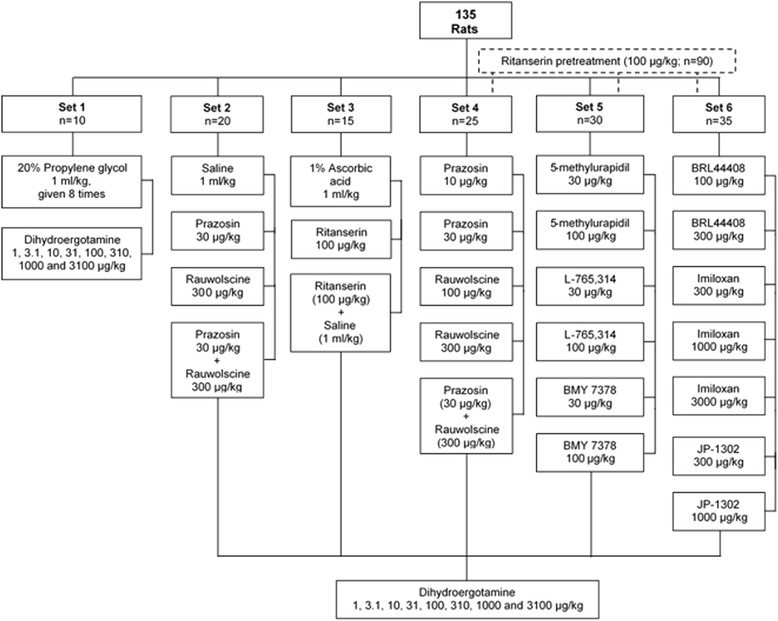
Number of pithed rats utilized in the six main sets and their subsequent division into the different groups (*n* = 5 each with no exception) used in the present study. The animals in set 1, set 2, and the first box of set 3 represent the control group animals (without ritanserin-pretreatment, *n* = 35). On the other hand, the second and third boxes of set 3, as well as sets 4, 5 and 6 represent the animals that were pretreated i.v. with 100 μg/kg ritanserin (*n* = 100)

Then, the 135 animals were divided into six main sets, namely, set 1 (*n* = 10), set 2 (*n* = 20), set 3 (*n* = 15), set 4 (*n* = 25), set 5 (*n* = 30) and set 6 (*n* = 35), as shown in Fig. 1. After the haemodynamic conditions were stable for at least 30 min, baseline values of diastolic blood pressure (a more accurate indicator of peripheral vascular resistance) and heart rate were determined. At this point, the effects produced by i.v. bolus injections of DHE (1, 3.1, 10, 31, 100, 310, 1000 and 3100 μg/kg; given cumulatively) on diastolic blood pressure and heart rate were investigated in animals with different pretreatments (see Fig. 1 and below for further details). In all cases, before eliciting the dose-response curves to DHE, a period of 10 min was allowed to elapse after the i.v. administration of antagonists or of their corresponding vehicles (given in a volume of 1 ml/kg); this period is appropriate for allowing drugs to interact with their corresponding receptors, as previously reported [10, 13].

Moreover, the cumulative dose-response curves to DHE were completed in about 50 min, and the intervals between the different doses of DHE (given in volumes of 1 ml/kg each) ranged between 4 and 7 min (as in each case we waited until the vasopressor response to the previous dose of DHE had reached a plateau). The same dose schedule was also applied for the vehicle of DHE (see below). The body temperature of each pithed rat (monitored with a rectal thermometer) was maintained at 37°C by a lamp.

### Experimental protocols

For the purpose of analysing the pharmacological profile of the receptors involved in the vasopressor responses to DHE, the six main sets of rats (as described above) were subsequently divided into different pretreatment groups (*n* = 5 each with no exception; see Fig. 1), for performing the following protocols.

### Protocol I. Effect of DHE on diastolic blood pressure

The first set of rats (*n* = 10; control animals with no pretreatment) was divided into two groups (*n* = 5 each) that received, as previously pointed out, i.v. bolus injections of: (i) 20% propylene glycol (vehicle of DHE, 1 ml/kg; given 8 consecutive times); and (ii) DHE (1, 3.1, 10, 31, 100, 310, 1000 and 3100 μg/kg). The effects produced by each dose of these compounds on diastolic blood pressure and heart rate were evaluated.

### Protocol II. Effect of α-adrenoceptor antagonists on DHE-induced vasopressor responses in control animals (non-pretreated with ritanserin)

The second set (*n* = 20, non-pretreated animals) was divided into four groups (*n* = 5 each) that received i.v. bolus injections of: (i) saline (vehicle of prazosin and rauwolscine, 1 ml/kg); (ii) 30 μg/kg prazosin; (iii) 300 μg/kg rauwolscine; and (iv) the combination of 30 μg/kg prazosin plus 300 μg/kg rauwolscine. After 10 min, a dose-response curve to DHE was elicited as previously described.

### Protocol III. Effect of ritanserin pretreatment on the DHE-induced vasopressor responses

The third set (*n* = 15), divided into 3 groups (*n* = 5 each), received i.v. bolus injections of: (i) 1% ascorbic acid (vehicle of ritanserin; 1 ml/kg); (ii) 100 μg/kg ritanserin; and (iii) 100 μg/kg ritanserin followed by 1 ml/kg physiological saline (vehicle of the α_1_- and α_2_-adrenoceptor antagonists). After 10 min, a dose-response curve to DHE was elicited as previously described. Considering the pretreatment of the last group (i.e. 100 μg/kg ritanserin plus 1 ml/kg physiological saline), the fourth, fifth and sixth sets were systematically pretreated with ritanserin (100 μg/kg, i.v.) and then with blocking doses of several α_1_- and α_2_-adrenoceptor antagonists (see Fig. 1) as follows.

### Protocol IV. Effect of α-adrenoceptor antagonists on DHE-induced vasopressor responses in ritanserin-pretreated animals

The fourth set (*n* = 25; pretreated with ritanserin), divided into five groups (*n* = 5 each), received i.v. injections of: (i) prazosin (10 μg/kg); (ii) prazosin (30 μg/kg); (iii) rauwolscine (100 μg/kg); (iv) rauwolscine (300 μg/kg); and (v) the combination prazosin (30 μg/kg) plus rauwolscine (300 μg/kg). Ten min later, a dose-response curve to DHE was elicited as described above.

### Protocol V. Effect of α_1_-adrenoceptor antagonists on DHE-induced vasopressor responses in ritanserin-pretreated animals

The fifth set (*n* = 30; pretreated with ritanserin), divided into six groups (*n* = 5 each), received i.v. injections of: (i) 5-methylurapidil (30 μg/kg); (ii) 5-methylurapidil (100 μg/kg); (iii) L-765,314 (30 μg/kg); (iv) L-765,314 (100 μg/kg); (v) BMY 7378 (30 μg/kg); and (vi) BMY 7378 (100 μg/kg). Ten min thereafter, a dose-response curve to DHE was elicited.

### Protocol VI. Effect of α_2_-adrenoceptor antagonists on DHE-induced vasopressor responses in ritanserin-pretreated animals

The sixth set (*n* = 35; pretreated with ritanserin), divided into seven groups (*n* = 5 each), received i.v. injections of: (i) BRL44408 (100 μg/kg); (ii) BRL44408 (300 μg/kg); (iii) imiloxan (300 μg/kg); (iv) imiloxan (1000 μg/kg); (v) imiloxan (3000 μg/kg); (vi) JP-1302 (300 μg/kg); and (vii) JP-1302 (1000 μg/kg). After 10 min, a dose-response curve to DHE was elicited.

### Data presentation and statistical evaluation

All data in the text and figures are presented as the means ± S.E.M. It is noteworthy that the data and statistical analysis used in the present study comply with the recommendations on experimental design and analysis in pharmacology, including that the data subjected to statistical analysis should have a minimum of *n* = 5 independent samples/individuals per group [19]. The changes on the baseline values of diastolic blood pressure and heart rate produced by i.v. bolus injections of DHE were determined after the administration of vehicles or antagonists. The difference between the changes in diastolic blood pressure within one subgroup of animals was evaluated with Student-Newman-Keul’s test, once a two-way repeated measures analysis of variance revealed that the samples represented different populations [20]. Statistical significance was accepted at *P* < 0.05.

### Drugs

Apart from the anaesthetic (sodium pentobarbital), the compounds used in the present study (obtained from the sources indicated) were: ritanserin; prazosin hydrochloride; rauwolscine hydrochloride; 5-methylurapidil; imiloxan hydrochloride; (2S)-4-(4-amino-6,7-dimethoxy-2-quinazolinyl)-2-[[(1,1-dimethylethyl)amino]carbonyl]-1-piperazinecarboxylic acid, phenylmethyl ester hydrate (L-765,314 hydrate); 8-[2-[4-(2-methoxyphenyl)-1-piperazinyl]ethyl]-8-azaspiro[4.5]decane-7,9-dione dihydrochloride (BMY 7378 dihydrochloride); propylene glycol (PPG); and L-ascorbic acid (Sigma Chemical Co., St. Louis, MO, U.S.A.); 2-[2H-(1-methyl-1,3-dihydroisoindole)methyl]-4,5-dihydroimidazole maleate (BRL44408 maleate) (Tocris Cookson Inc., Ellisville, MO, USA); acridin-9-yl-[4-(4-methylpiperazin-1-yl)-phenyl amine] hydrochloride (JP-1302 hydrochloride) (gift: Orion Corporation ORION PHARMA, Turku, Finland); and dihydroergotamine mesylate (gift: Novartis Pharma, Mexico City, Mexico). All compounds were dissolved in physiological saline. When needed, 1% ascorbic acid was used to dissolve ritanserin or 20% PPG (dissolved in bidistilled water) to dissolve DHE. Initially, DHE (3100 μg/ml) was dissolved in 20% PPG and the subsequent solutions were finally diluted with physiological saline. Fresh solutions were prepared for each experiment.

## Results

### Systemic haemodynamic variables

**Fig. 2:**
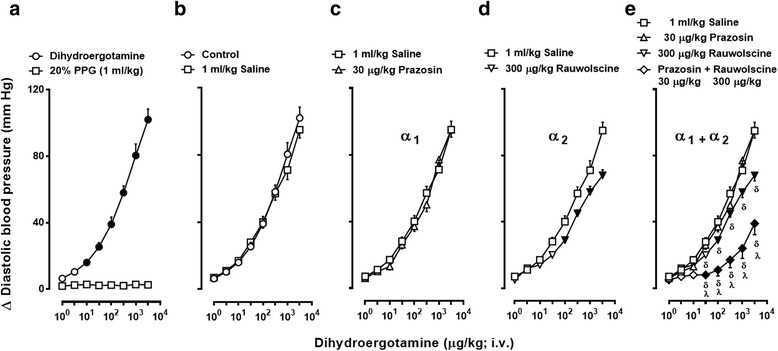
**a** Vasopressor responses produced by i.v. bolus injections of either dihydroergotamine (○; 1, 3.1, 10, 31, 100, 310, 1000 and 3100 μg/kg, given cumulatively) or equivalent volumes of 20% propylene glycol (☐; PPG, 1 ml/kg given 8 times consecutively). **b**-**e** Increases in diastolic blood pressure produced by i.v. dihydroergotamine (1–3100 μg/kg) after i.v. treatment with: (**b**) saline (☐, 1 ml/kg); (**c**) 30 μg/kg prazosin (△); (**d**) 300 μg/kg rauwolscine (▽); or (**e**) the combination of 30 μg/kg prazosin plus 300 μg/kg rauwolscine (**◇**) (*n* = 5 each). Solid symbols (●, ▼,◆) represent significantly different responses (*P* < 0.05) versus the corresponding volume of 20% PPG (☐), or versus the saline group (control, ☐), or versus the saline group (control, ☐) ^**δ**^ *P* < 0.05 versus 30 μg/kg prazosin (**e**). ^**λ**^ *P* < 0.05 versus 300 μg/kg rauwolscine (**e**). Note that the responses to DHE in the group pretreated with 1 ml/kg saline (**b**) is the same as that depicted in (**c**), (**d**) and (**e**), but they are illustrated here for comparative purposes. Moreover, for the sake of clarity when making comparisons, the responses produced after prazosin or rauwolscine in (**e**) are the same as those shown in (**c**) and (**d**). Data are shown as means ± SEM. Δ Diastolic blood pressure stands for “increases in diastolic blood pressure”

The baseline values of diastolic blood pressure and heart rate in the 135 pithed rats were 59 ± 2 mmHg and 230 ± 4 beats/min, respectively. These variables remained practically unchanged (*P* > 0.05; as compared with the corresponding untreated control group) in the groups of animals pretreated with all doses of the antagonists or their vehicles (see Table 2), as previously reported [10, 21]. In contrast, i.v. bolus injections of DHE (1, 3.1, 10, 31, 100, 310, 1000 and 3100 μg/kg; given cumulatively), but not of the corresponding volumes of vehicle (20% PPG, 1 ml/kg; given 8 times), produced dose-dependent vasopressor responses in untreated control animals (Fig. 2a).

**Table 2:** Values of DBP and HR before and 10 min after administration of compounds

Treatment	Doses (μg/kg)	Diastolic blood pressure (DBP) (mm Hg)		Heart rate (HR) (beats/min)	
		Before	After	Before	After
20% (*v*/v) propylen glycol	1^a^	55 ± 11	55 ± 12	200 ± 1	200 ± 1
Dihydroergotamine	3100	^b^	^b^	253 ± 45	285 ± 38
Saline^c^	1^a^	44 ± 5	44 ± 5	232 ± 11	232 ± 11
Prazosin^c^	30	42 ± 3	42 ± 3	234 ± 8	232 ± 9
Rauwolscine^c^	300	48 ± 3	51 ± 3	235 ± 11	244 ± 8
Prazosin+rauwolscine^c^	30 + 300	38 ± 3	41 ± 5	252 ± 10	234 ± 8
1% (*w*/*v*) ascorbic acid	1^a^	57 ± 3	60 ± 3	220 ± 12	220 ± 12
Ritanserin	100	70 ± 8	70 ± 8	193 ± 13	193 ± 13
Saline	1^a^	68 ± 10	65 ± 9	201 ± 26	203 ± 27
Prazosin	10 30	74 ± 11 61 ± 5	75 ± 10 61 ± 6	243 ± 8 183 ± 31	245 ± 17 183 ± 31
Rauwolscine	100 300	63 ± 5 51 ± 3	60 ± 5 47 ± 4	213 ± 7 238 ± 13	214 ± 8 240 ± 14
Prazosin + rauwolscine	30 + 300	51 ± 4	45 ± 5	218 ± 9	224 ± 14
5-methylurapidil	30 100	50 ± 7 54 ± 9	51 ± 6 52 ± 8	228 ± 15 232 ± 14	224 ± 12 236 ± 16
L-765,314	30 100	68 ± 8 64 ± 8	65 ± 8 63 ± 10	238 ± 20 226 ± 11	240 ± 16 226 ± 11
BMY 7378	30 100	68 ± 9 63 ± 3	66 ± 10 60 ± 3	240 ± 12 256 ± 12	238 ± 12 251 ± 13
BRL44408	100 300	65 ± 4 52 ± 8	65 ± 4 53 ± 9	232 ± 22 228 ± 13	228 ± 20 225 ± 15
Imiloxan	300 1000 3000	70 ± 10 69 ± 10 68 ± 6	69 ± 9 66 ± 11 66 ± 7	246 ± 9 247 ± 9 268 ± 16	248 ± 9 252 ± 7 271 ± 16
JP-1302	300 1000	58 ± 3 63 ± 7	58 ± 3 62 ± 10	247 ± 14 212 ± 10	278 ± 13 212 ± 10

Values are presented as means ± SEM

^a^1 ml/kg

**Fig. 3:**
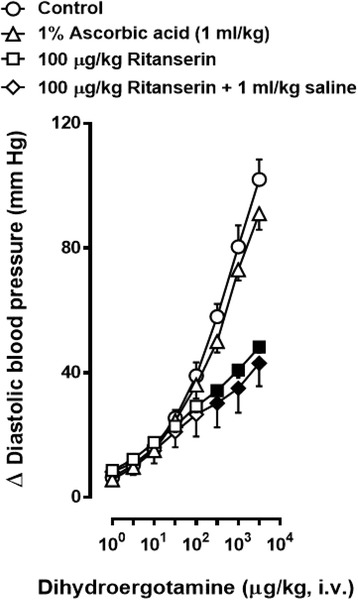
Increases in diastolic blood pressure produced by i.v. dyhydroergotamine (1–3100 μg/kg) after i.v. treatment with 1% ascorbic acid (△, 1 ml/kg); 100 μg/kg ritanserin (☐); or 100 μg/kg ritanserin followed by 1 ml/kg saline (**◇**) (*n* = 5 each). Empty symbols depict either control responses in untreated animals (**○**) or non-significant (*P* > 0.05) responses (△, ☐,**◇**) versus the control group (○). Solid symbols (■,◆) represent significantly different responses (*P* < 0.05) versus the control group (**○**) or versus the group pretreated with 1% ascorbic acid (△). Data are shown as means ± SEM. Δ Diastolic blood pressure stands for “increases in diastolic blood pressure”

**Fig. 4:**
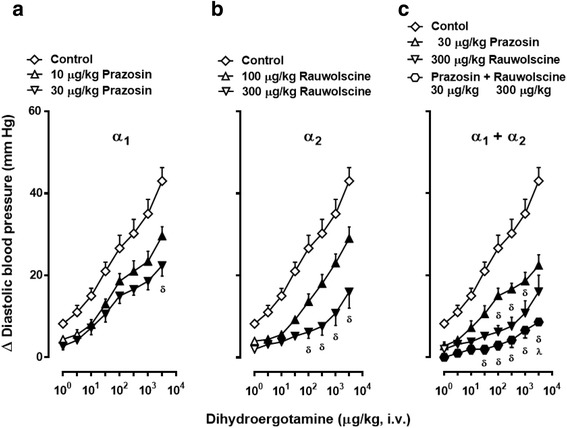
Increases in diastolic blood pressure produced by i.v. dihydroergotamine (1–3100 μg/kg) after i.v. treatment with: (**a**) prazosin; (**b**) rauwolscine; or (**c**) the combination of prazosin plus rauwolscine in animals pretreated (i.v.) with 100 μg/kg ritanserin. Empty symbols depict either: (i) the responses to DHE in the group pretreated with 100 μg/kg ritanserin followed by 1 ml/kg saline (**◇**, which represent the same data as those shown in Fig. 3, but it is illustrated here as the control for comparative purposes); or (ii) non-significant responses (△, ▽) versus control (**◇**). Solid symbols (▲,▼,) represent significantly different responses (*P* < 0.05) versus control (◇). ^**δ**^ *P* < 0.05 versus 10 μg/kg prazosin (**a**), 100 μg/kg rauwolscine (**b**) or 30 μg/kg prazosin (**c**). ^**λ**^ *P* < 0.05 versus 300 μg/kg rauwolscine (**c**). Note that the responses to DHE in the groups pretreated with either 30 μg/kg prazosin (**a**) or 300 μg/kg rauwolscine (**b**) are the same as those depicted in (**c**), but they are illustrated here for comparative purposes. Data are shown as means ± SEM. Δ Diastolic blood pressure stands for “increases in diastolic blood pressure”

**Fig. 5:**
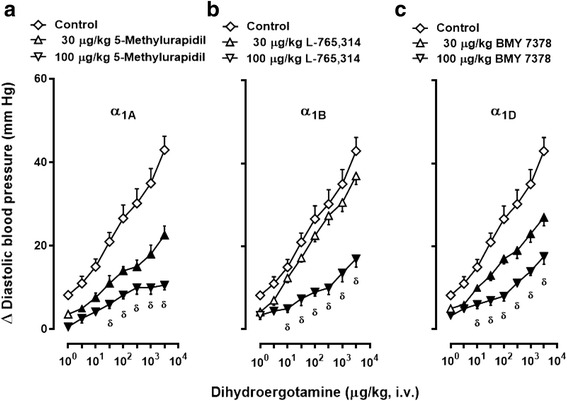
Increases in diastolic blood pressure by i.v. dihydroergotamine (1–3100 μg/kg) after i.v. treatment with: (**a**) 5-methylurapidil; (**b**) L-765,314; or (**c**) BMY 7378 in animals pretreated (i.v.) with 100 μg/kg ritanserin. Empty symbols depict either: (i) the responses to DHE in the group pretreated with 100 μg/kg ritanserin followed by 1 ml/kg saline (**◇**, which depicts the same data as those shown in Fig. 3, but it is illustrated here as the control for comparative purposes); or (ii) non-significant responses (△,▽) versus control (**◇**). Solid symbols (▲,▼) represent significantly different responses (*P* < 0.05) versus control (◇). ^δ^ *P* < 0.05 versus 30 μg/kg of: 5-methylurapidil (**a**), L-765,314 (**b**) or BMY 7378 (**c**). Data are shown as means ± SEM. Δ Diastolic blood pressure stands for “increases in diastolic blood pressure”

**Fig. 6:**
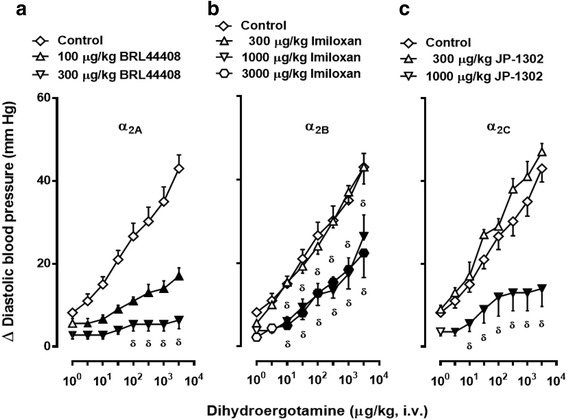
Increases in diastolic blood pressure by i.v. dihydroergotamine (1–3100 μg/kg) after i.v. treatment with: (**a**) BRL44408; (**b**) imiloxan; or (**c**) JP-1302 in animals pretreated (i.v.) with 100 μg/kg ritanserin. Empty symbols depict either: (i) the responses to DHE in the group pretreated with 100 μg/kg ritanserin followed by 1 ml/kg saline (**◇**, which depicts the same data as those shown in Fig. 3, but it is illustrated here as the control for comparative purposes); or (ii) non-significant responses (△, ▽,⎔ versus control (**◇**). Solid symbols (▲,▼,) represent significantly different responses (*P* < 0.05) versus control (◇). ^δ^ *P* < 0.05 versus 100 μg/kg BRL44408 (**a**), 300 μg/kg imiloxan (**b**) or 300 μg/kg JP-1302 (**c**). Data are shown as means ± SEM. Δ Diastolic blood pressure stands for “increases in diastolic blood pressure”

^b^These values were determined before and after 1, 3.1, 10, 31, 100, 310, 1000 and 3100 μg/kg DHE, and are shown in Figs. 2, 3, 4, 5, and 6

^c^These values were obtained from control rats (non-treated with ritanserin)

### Effects of vehicle or α-adrenoceptor antagonists on the vasopressor responses to DHE in animals without ritanserin-pretreatment

Figure 2 (b, c, d and e) shows that the vasopressor responses to DHE in pithed rats without ritanserin-pretreatment, which remained unchanged (*P* > 0.05) after i.v. administration of 1 ml/kg saline (vehicle of the α_1_- and α_2_-adrenoceptor antagonists; Fig. 2b) were: (i) resistant to blockade (*P* > 0.05) after 30 μg/kg prazosin (α_1_; Fig. 2c); (ii) slightly (though significantly) attenuated after 300 μg/kg rauwolscine (at 100, 310, 1000 and 3100 μg/kg DHE) (α_2_; Fig. 2d); and (iii) markedly blocked (*P* < 0.05) after the combination 30 μg/kg prazosin plus 300 μg/kg rauwolscine (at 31, 100, 310, 1000 and 3100 DHE) (Fig. 2e).

### Effects of vehicles or ritanserin on the vasopressor responses to DHE

Figure 3 illustrates that the vasopressor responses to DHE in the control (untreated) animals: (i) did not significantly differ from those elicited in the animals pretreated with 1% ascorbic acid (vehicle of ritanserin; 1 ml/kg, i.v.); and (ii) were significantly blocked at 310, 1000 and 3100 μg/kg DHE (whereas those produced by lower doses of DHE remained unaffected) in the animals pretreated with 100 μg/kg ritanserin or with 100 μg/kg ritanserin followed by 1 ml/kg saline (vehicle of the α_1_- and α_2_-adrenoceptor antagonists).

### Effects of vehicle or the α-adrenoceptor antagonists on the vasopressor responses to DHE in ritanserin-pretreated rats

Figures 4, 5 and 6 show that in ritanserin-pretreated animals, the vasopressor responses to DHE (as compared with vehicle-treated animals; control), were:

Figure 4) Significantly blocked (*P* < 0.05) in animals pretreated with the antagonists prazosin (α_1_, 10 and 30 μg/kg; Fig. 4a) or rauwolscine (α_2_, 100 and 300 μg/kg; Fig. 4b), with this blockade being dose-dependent and apparently more marked with rauwolscine. These results clearly contrast with those shown in Fig. 2d (see above). After treatment with the combination 30 μg/kg prazosin plus 300 μg/kg rauwolscine, the blockade of the response to 3100 μg/kg DHE was even more pronounced (*P* < 0.05) than that produced by each antagonist given individually (Fig. 4c).

Figure 5) Dose-dependently blocked in animals treated with 30–100 μg/kg of 5-methylurapdil (α_1A_; Fig. 5a) or BMY 7378 (α_1D_; Fig. 5c), and blocked only by 100 μg/kg of L-765,314 (α_1B_; Fig. 5b).

Figure 6) (i) Dose-dependently blocked in animals treated with 100–300 μg/kg BRL44408 (α_2A_; Fig. 6a); (ii) significantly attenuated, but not dose-dependently blocked, by 1000–3000 μg/kg imiloxan (α_2B_; Fig. 6b); and (iii) blocked only by 1000 μg/kg JP-1302 (α_2C_; Fig. 6c).

It is worthy of note that in Figs. 4, 5 and 6 the dose-response curve to DHE elicited in the group pretreated with 100 μg/kg ritanserin followed by 1 ml/kg saline (control) is the same as that shown in Fig. 3 but, for the sake of clarity, it was considered as a control for comparative purposes.

## Discussion

### General

In addition to the implications discussed below, our findings show that i.v. pretreatment with 100 μg/kg ritanserin (a dose devoid of α_1_-adrenoceptor blockade in pithed rats [15]) is a conditio sine qua non for demonstrating the blockade produced by prazosin alone (and the role of α_1_-adrenoceptors) in the DHE vasopressor responses. In keeping with this view: (i) in animals without ritanserin-pretreatment the vasopressor responses to DHE remained unchanged after 30 μg/kg prazosin (Fig. 2c), a dose that very potently blocks the α_1_-adrenoceptors mediating vasopressor responses in pithed rats [15]; and (ii) a component of these vasopressor responses (particularly at 310, 1000 and 3100 μg/kg DHE) is mediated by 5-HT_2_ receptors in view of the blockade produced by 100 μg/kg ritanserin (Fig. 3), whereas the ritanserin-resistant component is mediated by other receptors. In this respect, our findings showing that the remaining vasopressor responses to DHE after ritanserin-pretreatment were attenuated by 10 and 30 μg/kg prazosin (Fig. 4a) and that they were markedly blocked by 100 and 300 μg/kg rauwolscine (Fig. 4b) establish the involvement of rauwolscine-sensitive α_2_-adrenoceptors and, to a lesser extent, of prazosin-sensitive α_1_-adrenoceptors. In agreement with our findings, Roquebert and Grenié [6] reported that 500 μg/kg prazosin (i.v.) failed to block the vasopressor responses to DHE in pithed rats without pretreatment with a 5-HT_2_ receptor antagonist. Accordingly, this apparent failure by 30 μg/kg prazosin (Fig. 2c) or 500 μg/kg prazosin [6] implies that activation of vascular 5-HT_2_ receptors by higher doses of DHE, which displays a high affinity for 5-HT_2A_ receptors (pK_i_ = 8.54) [7], may have masked the blockade of α_1_-adrenoceptors by prazosin. Certainly, prazosin has higher affinity (approximately 1 to 2 logarithmic units) than DHE for α_1_-adrenoceptors (Table 1). However, the affinity (pK_i_) of prazosin for 5-HT_2_ receptors (if any) is <<4 [22], whereas that of DHE is 8.54 (see above). Therefore, it is highly unlikely that prazosin is blocking 5-HT_2_ receptors. This suggestion is reinforced when considering that the blockade produced by the combination 30 μg/kg prazosin plus 300 μg/kg rauwolscine in the absence of ritanserin was more pronounced than that produced by rauwolscine alone (Fig. 2e). This line of reasoning can also account for the higher potency of blockade by rauwolscine in ritanserin-pretreated rats (Fig. 4b) as compared to that in animals without ritanserin pretreatment (Fig. 2d). These findings, taken together, may suggest that DHE-induced vasopressor responses involve the sum of a combination of effects mediated by activation of 5-HT_2A_ receptors, α_1_-adrenoceptors and α_2_-adrenoceptors.

In addition, our experimental approach with ritanserin pretreatment further suggests that the vasopressor responses to DHE could be mainly mediated by α_1_- (probably α_1A_, α_1B_ and α_1D_) and α_2_- (probably α_2A_, α_2B_ and α_2C_) adrenoceptors, although some caution should be exerted when interpreting the “subtype selectivity” of the compounds used (see below and Table 1), as these responses were blocked by the antagonists: (i) 5-methylurapidil (α_1A_), L-765,314 (α_1B_) or BMY 7378 (α_1D_) (Fig. 5); and (ii) BRL44408 (α_2A_), imiloxan (α_2B_) or JP-1302 (α_2C_) (Fig. 6).

### Systemic haemodynamic variables

Our results in pithed rats show that DHE (administered cumulatively) produced dose-dependent increases in diastolic blood pressure (Fig. 2a) without significantly affecting heart rate (Table 2), as previously reported [6, 10, 21]. In this respect, since the central nervous system is not operative in pithed rats (see General methods section), the influence of central baroreflex mechanisms can be categorically excluded. Moreover, DHE was administered cumulatively because it produced sustained and long-lasting vasopressor responses, which may be due to the slow dissociation of the drug-receptor complex [23, 24]; however, our study provides no evidence whatsoever to support this view. Additionally, the baseline values of diastolic blood pressure and heart rate remaining practically unchanged by the α-adrenoceptor antagonists (Table 2) imply that their effects on the responses to DHE are: (i) unrelated to cardiovascular changes or physiological antagonism; and (ii) mediated by the direct interaction with its corresponding receptor. On the other hand, the difference in the baseline values of diastolic blood pressure in the different groups of animals (Table 2) may be attributed to biological variability, as observed in previous studies [10, 11, 15].

### Involvement of α_1_-and α_2_-adrenoceptors in the vasopressor responses to DHE

DHE displays affinity for a wide variety of receptors [1], with the same nanomolar affinity for rat α_1_-adrenoceptors (pK_i_: 8.0) and rat α_2_-adrenoceptors (pK_i_: 8.0) [7]. Interestingly, DHE can also interact with all α_1_- and α_2_-adrenoceptor subtypes (see Table 1). These findings may help explain, within the context of our study, the complex interactions of DHE. Within the bounds of adrenergic mechanisms in our study using ritanserin-pretreated rats, the functional role of α_1_- and α_2_-adrenoceptors in the vasopressor responses to DHE is clearly established, as these responses were: (i) blocked by prazosin (10–30 μg/kg; Fig. 4a) or by rauwolscine (100–300 μg/kg; Fig. 4b); and (ii) further blocked (particularly the response to 3100 μg/kg DHE) by the combination of prazosin plus rauwolscine (Fig. 4c). Certainly, in pithed rats, 30 μg/kg prazosin and 300 μg/kg rauwolscine are doses high enough to completely block the vasopressor responses mediated by, respectively, α_1_-adrenoceptors [15] and α_2_-adrenoceptors [13]. Nonetheless, there were some important differences in the profile of blockade produced by these antagonists. Indeed, the partial blockade of the DHE responses by 30 μg/kg prazosin, being slightly more pronounced than that produced by 10 μg/kg prazosin (Fig. 4a) may suggest that it was already a supramaximal dose that, in addition to completely blocking α_1_-adrenoceptors, could have weakly blocked α_2_-adrenoceptors (particularly the α_2B_ and α_2C_-adrenoceptor subtypes, for which it displays a moderate affinity; Table 1). In contrast, the marked blockade by 300 μg/kg rauwolscine, being more pronounced than that by 100 μg/kg rauwolscine (Fig. 4b), may suggest (although does not directly prove) a major role of α_2_-adrenoceptors (as compared to α_1_-adrenoceptors). This suggestion may help partly explain why Roquebert and Grenié [6] could show the role of α_2_-adrenoceptors, but not of α_1_-adrenoceptors, in the DHE responses in Wistar rats without 5-HT_2_ receptor blockade. Admittedly, Roquebert and Grenié [6]: (i) did not analyse the effects of the combination prazosin + yohimbine as we did with the combination prazosin plus rauwolscine in animals without ritanserin pretreatment (Fig. 2e); and (ii) used older rats (300–350 g) anaesthetised with ether. Certainly, the functional expression of rat vascular α_1_-adrenoceptor subtypes depends on several factors, including age [25].

Interestingly, the failure of the combination prazosin plus rauwolscine to abolish (although markedly blocked) the DHE responses in ritanserin-pretreated rats (Fig. 4c) cannot categorically exclude the possible role of additional (although negligible) mechanisms, including an enhanced synthesis of proconstrictor prostaglandins by DHE, as reported by Müller-Schweinitzer [26].

### The possible role of the different α_1_- and α_2_-adrenoceptor subtypes in the responses to DHE

As suggested above, the vasopressor responses to DHE in ritanserin-pretreated rats are mainly mediated by rauwolscine-sensitive α_2_-adrenoceptors and, apparently to a lesser extent, by prazosin-sensitive α_1_-adrenoceptors. Nevertheless, these antagonists do not display selective affinities for distinguishing amongst their corresponding α_1_- and α_2_-adrenoceptor subtypes (Table 1). Hence, the effects of relatively more selective antagonists for the α_1_-adrenoceptor subtypes (i.e. 5-methylurapidil [α_1A_], L-765,314 [α_1B_] and BMY 7378 [α_1D_]) and the α_2_-adrenoceptor subtypes (i.e. BRL44408 [α_2A_], imiloxan [α_2B_] and JP-1302 [α_2C_]) (Table 1) were further investigated in an attempt to identify the subtypes involved.

The fact that the DHE responses were blocked after administration of each of these antagonists for α_1_- (Fig. 5) and α_2_-adrenoceptors (Fig. 6) basically suggests the involvement of, respectively, the α_1A_/α_1B_ /α_1D_ subtypes and the α_2A_/α_2B_/α_2C_ subtypes. Importantly, the doses used of these antagonists have previously been shown: (i) to completely block the vasopressor responses mediated by the α_1A_/α_1B_/α_1D_ subtypes and the α_2A_/α_2B_/α_2C_ subtypes in pithed rats [10, 13]; and (ii) to correlate with the affinities for their respective subtypes [27] (see Table 1). Notwithstanding, the differences in the profile of blockade produced by each of the above antagonists deserve further considerations.

On the one hand, 30–100 μg/kg of 5-methylurapidil (Fig. 5a) and BMY 7378 (Fig. 5c) dose-dependently blocked the DHE responses and display very high affinity for, respectively, the α_1A_ (pK_i_: 9.0) and α_1D_ (pK_i_: 9.0) subtypes, but they also display moderate affinity for the other α_1_ subtypes (with pK_i_’s between 7.0 and 8.0; Table 1). Hence, one could imply that the high potency of these antagonists to block the DHE responses may be due to a marked blockade of their receptors, with partial blockade of the other α_1_ subtypes. However, Zhou and Vargas [28] showed in pithed rats that: (i) 500 μg/kg 5-methylurapidil blocked the vasopressor responses to the α_1A_-adrenoceptor agonist (R)A-61603; and (ii) 100–1000 μg/kg BMY 7378, which dose-dependently blocked the vasopressor responses to phenylephrine, failed to block those to (R)A-61603. Thus, it would seem logical to suggest that 5-methylurapidil (Fig. 5a) and BMY 7378 (Fig. 5c) are reasonably selective for blocking the α_1A_- and α_1D_-subtypes, respectively, as suggested by Willems et al. [27]. In contrast, the fact that only 100 μg/kg L-765,314 significantly blocked the DHE responses (Fig. 5b): (i) apparently matches with its slightly lower -but still high- affinity (pK_i_: 8.3) for the α_1B_ subtype and its moderate affinity for the α_1D_ subtype (Table 1); and (ii) implies a minor role of the α_1B_ subtype (relative to that of the α_1A_- and α_1D_- subtypes) in the systemic vasculature, as suggested by Daly et al. [29].

On the other hand, as to the role of the α_2_-adrenoceptor subtypes, BRL44408 and JP-1302 are “relatively selective” for, respectively, the α_2A_ (pK_i_: 8.7) and α_2C_ (pK_i_: 7.6) subtypes (Table 1). Thus, the high potency of BRL44408 (100–300 μg/kg; Fig. 6a) and the lower potency of JP-1302 (only at 1000 μg/kg; Fig. 6c) to block the DHE responses might suggest a major role of the α_2A_ subtype and a less predominant role of the α_2C_ subtype mediating vasopressor responses, as suggested by Gavin and Docherty [30]. However, the affinities of these antagonists for the α_1_- adrenoceptor subtypes have not been determined (Table 1). Interestingly, in pithed rats (*n* = 5), the vasopressor responses to i.v. bolus injections of 0.1, 0.3, 1, 3, 10 and 30 μg/kg phenylephrine (14 ± 2, 19 ± 2, 24 ± 2, 39 ± 5, 66 ± 7 and 115 ± 7 mmHg, respectively): (i) remained unaltered after an i.v. bolus of 100 μg/kg BRL44408 (16 ± 2, 20 ± 2, 25 ± 3, 40 ± 6, 69 ± 9 and 107 ± 12 mmHg); and (ii) were attenuated (at the highest doses) after an i.v. bolus of 300 μg/kg BRL44408 (12 ± 2, 12 ± 1, 18 ± 3, 29 ± 7, *54 ± 12 and *93 ± 16 mmHg; *P < 0.05) (unpublished observations). The latter finding may explain why the blockade produced by BRL4408 (Fig. 6a): (i) did not significantly differ (*P* > 0.05) from that produced by the combination prazosin plus rauwolscine (Fig. 4c); and (ii) was more pronounced than that produced by rauwolscine alone (Fig. 4b). In contrast, the affinity of imiloxan for the α_1_-adrenoceptor subtypes is very low (pK_i_ < 4; which excludes its interaction with these receptors), but its affinity for the α_2B_ (pK_i_: 7.3) and α_2C_ (pK_i_: 6.0) subtypes (Table 1) leaves very little room for in vivo selectivity, particularly at the doses used (Fig. 6b). Indeed, the blockade of the DHE responses by 1000 and 3000 μg/kg imiloxan being practically identical (Fig. 6b) seems to suggest a minor role of the α_2B_ (and probably also of the α_2C_) adrenoceptor subtype. Hence, we considered it unnecessary to explore the effect of more antagonist combinations.

Clearly, the above findings cannot be simply explained in terms of pure antagonism at a single receptor subtype in view of: (i) the nature of our pithed rat model (in which we cannot reach equilibrium conditions, nor can we categorically exclude the role of pharmacokinetic factors); (ii) the relative “selectivity” of the antagonists used (determined in vitro; Table 1); and (iii) the limited selectivity of these compounds when given i.v. in pithed rats.

### Potential clinical implications of the present results

Admittedly, the relative “selectivity” of the α_1_- and α_2_-adrenoceptor antagonists used in this study (see Table 1) would seem rather limited in view of the i.v. (systemic) administration of compounds and the additional role of pharmacokinetic factors (which cannot be completely ruled out in pithed rats). Consistent with these views, other studies performed in vivo with these compounds have also shown limited selectivity [31]. Notwithstanding, the pithed rat model is predictive of (cardio)vascular side effects [11, 12] and provides information that cannot be obtained from in vitro studies [32]. Moreover, from a clinical perspective, our findings may help understand the pharmacological profile of the adverse vascular side-effects (i.e. systemic vasoconstriction) produced by DHE (present results) and ergotamine [10], even when the pharmacological profile of the α-adrenoceptor subtypes mediating systemic vasoconstriction in rodents and humans is not identical [25].

On the other hand, although the vasoconstrictor responses to DHE mediated by α_1_- and α_2_-adrenoceptors are less pronounced (i.e. after ritanserin pretreatment; compare Fig. 3 with Figs. 4, 5 and 6), their effects gain importance in view of the long-lasting vasoconstriction induced by DHE, as previously reported [23, 24]. These findings are even more relevant from a clinical perspective in view of the already increased cardiovascular risk in migraine patients [33, 34]. Certainly, there are other drugs for the acute treatment of migraine [2, 35, 36], including the triptans (which produce selective cranial vasoconstriction) and calcitonin gene related peptide (CGRP) receptor antagonists and antibodies (which block the cranial vasodilatation produced by trigeminal release of CGRP). Regarding CGRP receptor antagonists and antibodies, they are clearly devoid of direct vasoconstrictor effects; notwithstanding, since CGRP may play a vasodilator protective role during ischemic (cerebral and cardiac) events, CGRP blockade could transform transient ischemic events into lethal infarcts [36]. Thus, the pharmacological analysis of the systemic vasoconstriction induced by the classical antimigraine agent DHE is of particular relevance for the further development of antimigraine drugs devoid of direct, as well as indirect, vascular side effects.

## Conclusion

Our results suggest that the vasopressor (systemic vasoconstrictor) responses to DHE in ritanserin pretreated pithed rats could be mediated by activation of α_2_ (probably α_2A_, α_2B_ and α_2C_)-adrenoceptors and, apparently to a lesser extent, by α_1_ (probably α_1A_, α_1B_ and α_1D_)-adrenoceptors. Admittedly, this conclusion is based on the assumption that all antagonists used are relatively selective (as deducted from in vitro binding data under equilibrium conditions; Table 1) for blocking their corresponding α-adrenoceptor (sub)types at the doses used in the present study.

## Abbreviations

DHE: Dihydroergotamine
i.p.: Intraperitoneal
i.v.: Intravenous
PPG: Propylene glycol
